# Unveiling the Multiradical Character of the Biphenylene Network and Its Anisotropic Charge Transport

**DOI:** 10.1021/jacs.2c02178

**Published:** 2022-04-27

**Authors:** Isaac Alcón, Gaetano Calogero, Nick Papior, Aleandro Antidormi, Kenan Song, Aron W. Cummings, Mads Brandbyge, Stephan Roche

**Affiliations:** † Catalan Institute of Nanoscience and Nanotechnology (ICN2), CSIC and BIST, Campus UAB, Bellaterra, Barcelona 08193, Spain; ‡ Institut für Chemie und Biochemie, Physikalische und Theoretische Chemie, Freie Universität Berlin, Arnimallee 22, Berlin 14195, Germany; § CNR Institute for Microelectronics and Microsystems (CNR-IMM), Zona Industriale, Strada VIII, 5, Catania 95121, Italy; ∥ Computing Center, Technical University of Denmark, Kongens Lyngby DK-2800, Denmark; ⊥ Physical Science and Engineering Division, King Abdullah University of Science and Technology (KAUST), Thuwal 23955, Saudi Arabia; # Department of Physics, Technical University of Denmark, Kongens Lyngby DK-2800, Denmark; ¶ Center for Nanostructured Graphene (CNG), Kongens Lyngby DK-2800, Denmark; ∇ ICREA-Institució Catalana de Recerca i Estudis Avançats, Barcelona 08070, Spain

## Abstract

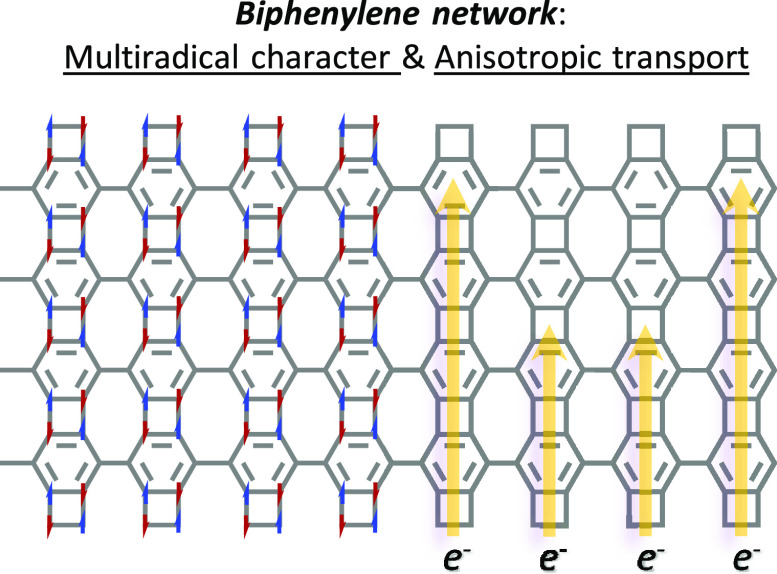

Recent progress in the on-surface synthesis and characterization of nanomaterials is facilitating the realization of new carbon allotropes, such as nanoporous graphenes, graphynes, and 2D π-conjugated polymers. One of the latest examples is the biphenylene network (BPN), which was recently fabricated on gold and characterized with atomic precision. This gapless 2D organic material presents uncommon metallic conduction, which could help develop innovative carbon-based electronics. Here, using first principles calculations and quantum transport simulations, we provide new insights into some fundamental properties of BPN, which are key for its further technological exploitation. We predict that BPN hosts an unprecedented spin-polarized multiradical ground state, which has important implications for the chemical reactivity of the 2D material under practical use conditions. The associated electronic band gap is highly sensitive to perturbations, as seen in finite temperature (300 K) molecular dynamics simulations, but the multiradical character remains stable. Furthermore, BPN is found to host in-plane anisotropic (spin-polarized) electrical transport, rooted in its intrinsic structural features, which suggests potential device functionality of interest for both nanoelectronics and spintronics.

## Introduction

**Figure 1:**
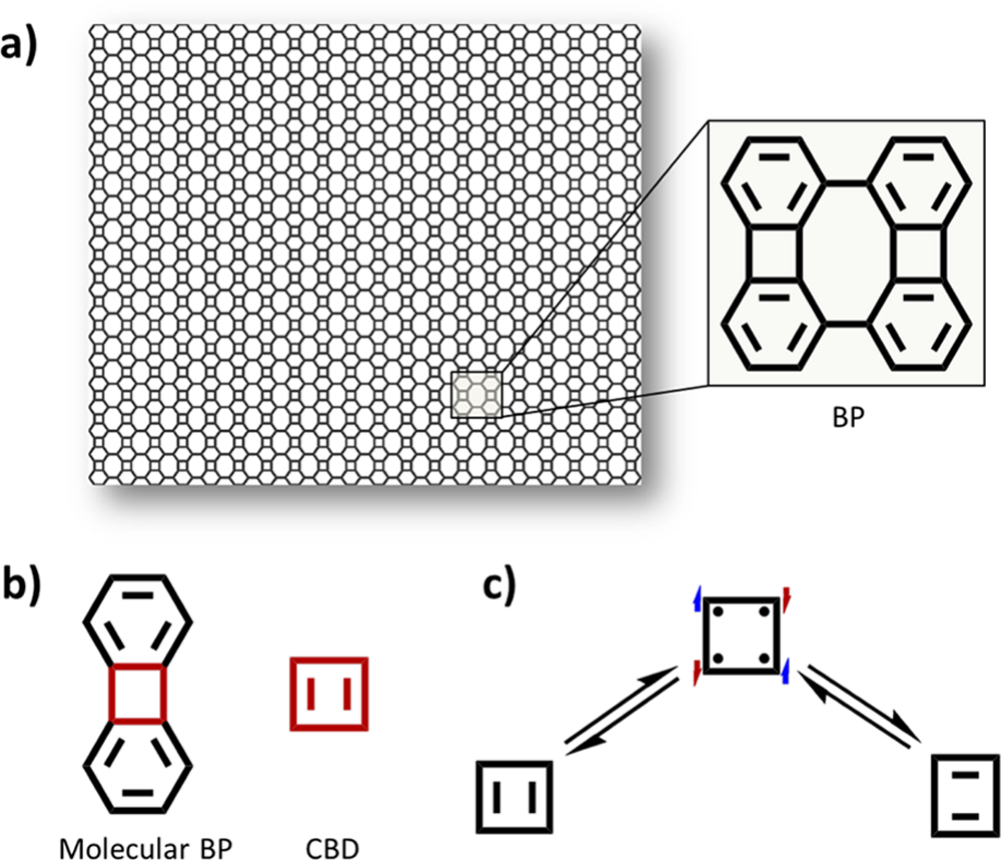
(a) BPN structure with an enlarged schematic view of its basic repeating unit. (b) Chemical structure of molecular BP, where the square ring resembling CBD is highlighted in red. (c) Lewis resonance forms of CBD: the two energetically degenerate closed-shell rectangular conformations are connected through an open-shell (radical) transition-state with a square conformation.

Graphene, the two-dimensional (2D) form of sp^2^ carbon,^1^ has catalyzed a pursuit for other 2D carbon allotropes, such as phagraphene,^2,3^ graphane,^4^ graphynes,^5−7^ graphdyines,^8,9^ grazynes,^10,11^ and 2D π-conjugated polymers.^12−16^ Interestingly, some of these materials have been experimentally synthesized, confirming, in particular cases, previously predicted electronic features such as semiconductor behavior or antiferromagnetism.^9,16−19^ The biphenylene network (BPN) is the latest member of the 2D carbon allotrope family to be experimentally reported.^20^ As depicted in Figure 1a, it is made of a combination of 4-, 6-, and 8-membered rings. Using scanning tunneling spectroscopy measurements,^20^ a closing of the band gap was found upon increasing the width of biphenylene ribbons, hence constituting a truly metallic 2D organic material. On the theoretical side, while initial simulations predicted BPN to be a semiconductor with a band gap of ca. 2 eV,^21^ more recent theoretical studies show BPN to be metallic,^22,23^ in line with experimental findings.^20^

More recently, other theoretical studies have scrutinized additional properties of BPN, such as its hydrogenation^24^ and lithiation capacity,^25^ or the anisotropic character of thermal transport within the 2D network.^26^ However, other fundamental aspects, such as the electrical transport characteristics of BPN or the effect of finite temperature on its electronic structure, essential for practical purposes, remain to be established.

In this work, we explore such aspects by combining first principles simulations with real-space quantum transport calculations. First, using density functional theory (DFT) simulations, the effect of finite temperature on BPN’s electronic structure is analyzed. Importantly, the calculations reveal the existence of a spin-polarized multiradical electronic state stable to thermal fluctuations, which could have important implications for the material chemical reactivity in practical use conditions (e.g., in air). Interestingly, this electronic solution leads to a band gap opening, though this result strongly depends on the utilized DFT functional. Second, by studying the injection of electronic currents in large-scale BPN samples, unidirectional charge transport conduction is observed, in striking contrast to other 2D hexagonal carbon allotropes, such as graphene.^27,28^ Transport simulations of the multiradical electronic solution of BPN reveal a spin-filtering effect with potential for spin devices. Overall, our study provides new insights into the electronic structure of BPN with implications for its future technological exploitation.

## Results and Discussion

### On the Multiradical Character of BPN

The term biphenylene (BP) was first used for the molecular analogue composed of two doubly connected phenyl rings,^29,30^ as represented in Figure 1b (molecular BP). This connection leads to a four-membered ring between the two phenyl rings, which resembles the cyclobutadiene structure (CBD in Figure 1b). CBD is a compound with two energetically degenerate closed-shell rectangular conformations,^31,32^ as depicted in Figure 1c. It was shown, both via theoretical calculations^33,34^ and more recently via electron paramagnetic resonance measurements,^35^ that the transition state between the two is of radical character (Figure 1c). This was demonstrated by the detection of spin-polarized triplet states populated at high temperatures,^35^ which is also in line with the high reactivity of CBD and the need to utilize protecting groups to isolate and characterize it.^36^

Therefore, the presence of “CBD-like” four-membered rings in BPN could indicate the existence of low-lying multiradical electronic solutions. Such states have not yet been reported for the 2D material, but there is some indication of their existence in the analogous 0D and 1D systems. Specifically, the non-bonding character of four-membered rings in molecular BP was demonstrated via molecular electrostatic potential topology calculations.^37^ Additionally, a recent study predicted the appearance of an antiferromagnetically aligned multiradical state in 1D BP, although this magnetic solution could not be detected in the on-surface synthesized material,^38^ potentially due to electron doping by the metallic surface.

To evaluate the possible existence of such multiradical states in BPN, we optimized the atomic and electronic structure of the 2D material with DFT calculations. These calculations utilize periodic boundary conditions and the hybrid HSE06^39,40^ functional within the generalized gradient approximation (GGA). We note that other GGA functionals with a different degree of Hartree Fock exchange (HFE) have also been tested (see the Experimental Section).

**Figure 2:**
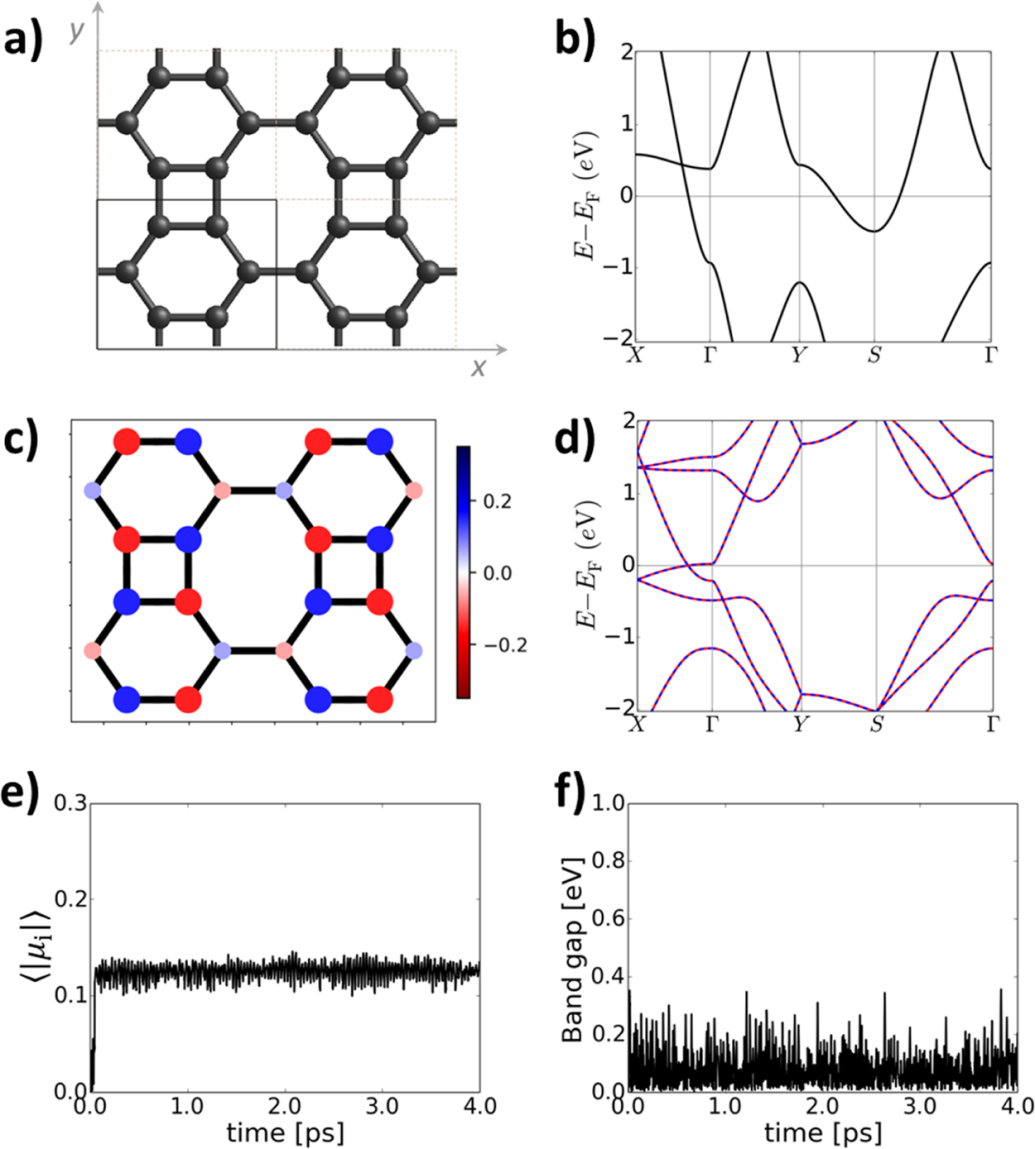
(a) Optimized atomic structure of BPN using the HSE06 DFT functional. A 2 × 2 supercell is shown, with the primitive unit cell indicated with a black square. (b) Electronic band structure of the metallic state in BPN using the primitive unit cell. (c) Atomically resolved spin population map of the multiradical state in the 2 × 2 BPN supercell (spin-up: blue and spin-down: red). The size and color intensity are proportional to the spin population value. (d) Multiradical state band structure (spin-up: blue dashed line and spin-down: red dashed line). Note that spin-up/spin-down bands are perfectly superimposed. (e) Average of the absolute atomically partitioned spin population, ⟨|μ_i_|⟩, during 4 ps of an ab initio molecular dynamics simulation at 300 K using the HSE06 functional and (f) associated electronic band gap.

Figure 2a shows the optimized atomic structure of BPN, where the primitive unit cell is indicated with a square. The resulting band structure, as shown in Figure 2b, indicates a metallic nature of the 2D material, in line with prior theoretical results^22,23,25^ and experimental findings.^20^ As has been previously shown for semimetallic 2D π-conjugated polymers,^12^ to capture any low-lying multiradical states with periodic DFT calculations, it is necessary to choose an antiferromagnetic (AFM) initial guess.^13,41^ As shown in Figure 2c, upon doing so, we obtain a multiradical state with an AFM spin alignment. This multiradical character emerges from the square CBD-like units, in agreement with the radical nature of this organic compound^35^ and recent studies on 1D BP.^38^ We note that this electronic state cannot be hosted in the minimal cell (square, as shown in Figure 2a), so to obtain it, we utilized the 2 × 2 supercell, as shown in Figure 2a. More details about the importance of the unit cell choice are provided in Figure S1 and Table S1 of Supporting Information.

The band structure of this multiradical state is shown in Figure 2d (a comparison with that of the non-spin-polarized metallic state for the analogous supercell can be seen in Figure S2 of Supporting Information). Figure 2d suggests that the multiradical state is still metallic, which is in agreement with experimental findings reporting a vanishing band gap for increasingly wider BP ribbons.^20^ However, the nature of this multiradical electronic solution appears to strongly depend on the percentage of HFE, and other hybrid functionals with higher HFE, such as PBE0,^42^ yield a band gap opening and a semiconducting state (see Figure S3). As shown in Table 1, this multiradical solution is the ground state of the system, being −0.22 eV below the non-spin-polarized metallic solution. This value depends on the utilized DFT flavor, but both tested functionals predict the multiradical configuration as the most stable electronic solution. On the other hand, the average of the absolute value of atomic spin populations, ⟨|μ_i_|⟩, which can be used as a measure of multiradical character,^41^ is very similar for both tested theoretical approaches (Table 1). In spite of the significant energy difference with the metallic state, such multiradical electronic solution has not been previously predicted for BPN. As explained, this may be related to the inability of DFT to capture such a spin-polarized solution without a proper spin-polarized initial guess and the need to use a supercell (e.g., 1 × 2, 2 × 2, or larger) hosting such AFM alignment of spins (see Figure S1).

**Table 1:** Energy Difference between the Multiradical (Rad) and the Closed-Shell Metallic (Met) Electronic Solutions, Average of the Absolute Value of Atomic Spin Populations in the Multiradical State (⟨|μ_i_|⟩) and Associated Electronic Band gaps as Calculated with Either HSE06 or PBE0 DFT Functionals, Considering 2 × 2 Supercells^a^

	HSE06	PBE0
*E*_Rad_ – *E*_Met_ (meV)	–223.8	–399.1
⟨|μ_i_|⟩	0.12	0.14
band gap (eV)	0.00	0.23

a See the methods section for details.

In order to study the effect of thermal fluctuations on the electronic configuration of BPN, we employ ab initio molecular dynamics simulations (AIMDS) at 300 K at the same level of theory (see the methods section for details). In this case, an initial spin guess of 0 is used, to avoid any bias of the system toward the spin-polarized multiradical solution. As shown in Figure 2e, we plot the time dependence of ⟨|μ_i_|⟩ during the molecular dynamics simulation, which evidences the spontaneous appearance of a spin-polarized multiradical character that remains stable throughout the entire simulation run and stays similar to the value of ⟨|μ_i_|⟩ at 0 K (Table 1). A similar situation is found when using the PBE0 functional (see Figure S4). However, in agreement with our results at 0 K (Table 1), the electronic band gap varies with the DFT functional. The HSE06 functional predicts a minor gap opening below 0.1 eV during the AIMDS (Figure 2f), whereas PBE0 leads to a steady band gap of ca. 0.45 eV (Figure S4). The increase in these values with respect to those at 0 K (Table 1) may originate from electronic localization caused by thermal vibrations. These results suggest that the material’s band gap could significantly change with increasing temperature, potentially leading to a metal → semiconductor transition. Further experimental characterization would be of high interest to clarify this point, or whether BPN exhibits thermally activated band gap fluctuations.

In any case, in spite of the highlighted discrepancies between the different tested functionals, one may conclude that the multiradical state in BPN is stable at finite temperature, which is consistent with the 0 K results (Table 1) that show that this is the ground state of the system, well below the non-spin-polarized solution. Radical or multiradical organic (carbon-based) systems are known to be highly chemically reactive species,^43,44^ thus requiring a particular chemical functionalization to protect the radical centers (e.g., via sterical hindrance^45^). Since BPN is a planar material, our predictions suggest that it might become a highly chemically reactive system under practical use conditions (e.g., at finite temperature in air) and implies a potential need to encapsulate it (e.g., between inert hexagonal boron–nitride layers^46^) in order to stabilize it for use in solid-state electronic devices. Finally, such multiradical nature should make BPN prone to charge-transfer effects when in contact with metallic substrates, such as recently demonstrated for fully planar organic radical compounds deposited on Au or Ag.^47^ As it may be seen in Figure S5 in Supporting Information, doping the material with holes or electrons may lead to the full depletion of its spin-polarized multiradical character (making it a closed shell), which is in line with recent findings with 1D BP.^38^ This highlights the need to transfer the 2D carbon material to insulating surfaces, such as NaCl (regularly used for graphene nanoribbons^48,49^), in order to detect the multiradical nature herein predicted, and to be able to measure its transport properties (see below).

### On the Anisotropic Charge Transport of BPN

Contrary to graphene and other hexagonal carbon allotropes, BPN has an atomic periodic structure, which is anisotropic in the *x*–*y* plane. Specifically, the six-membered rings are connected via single C–C bonds along the *x*-direction, whereas they are connected via two C–C bonds along the *y*-direction (see Figure 2a) which form the CBD-like ring. It has already been shown, via large-scale transport simulations, that structurally anisotropic carbon 2D materials lead to anisotropic electronic transport, such as in the case of nanoporous graphenes^50−52^ or in the so-called grazynes.^10,11^ A recent study of BPN has also predicted that phonons (i.e., thermal energy) are transported differently along the two in-plane directions.^26^ However, till date, the electronic transport characteristics of this novel 2D material remain to be investigated.

**Figure 3:**
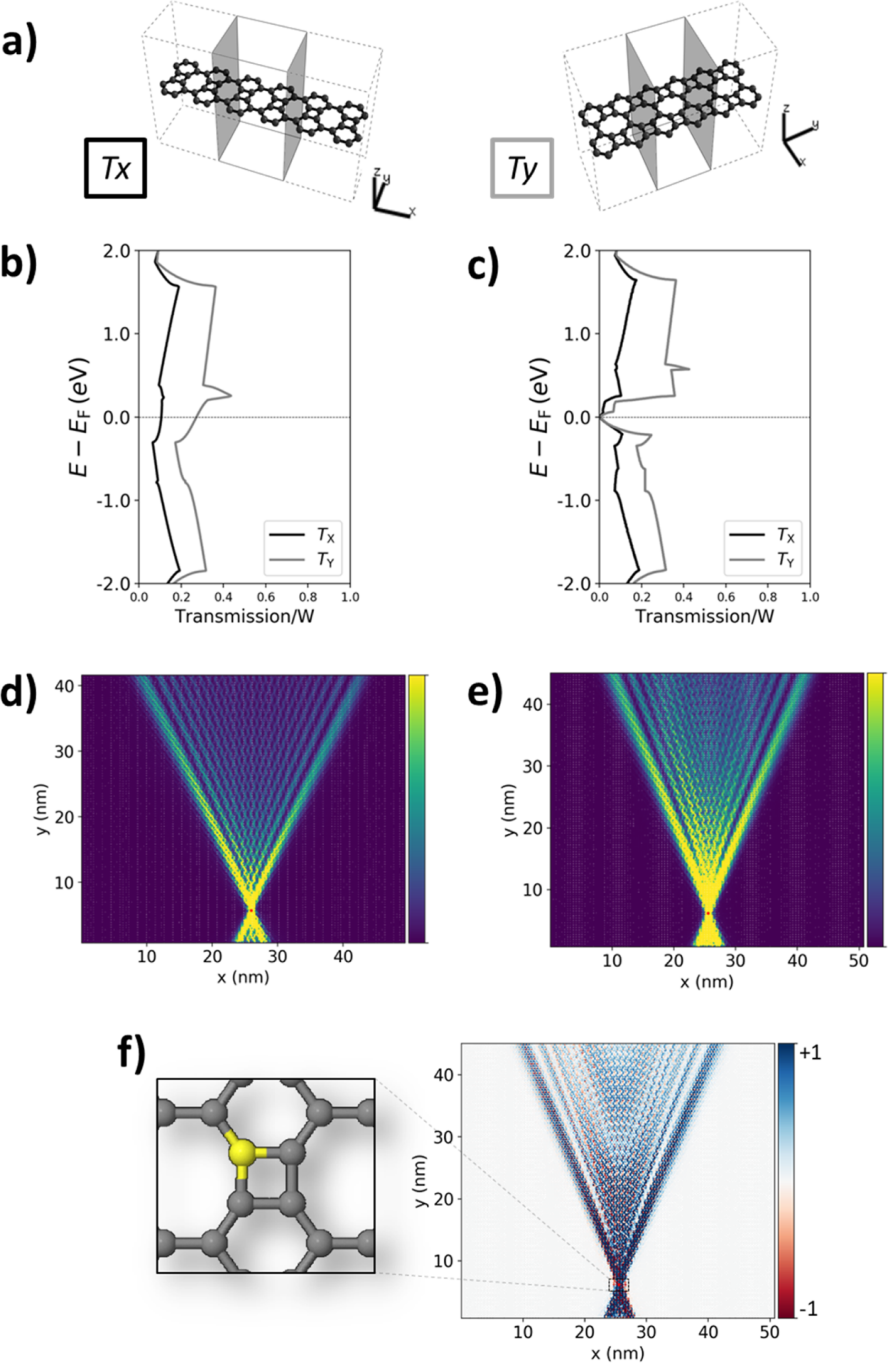
(a) Basic transport setup to calculate electronic transmission along the *x*-direction (left panel) and *y*-direction (right-panel) in BPN. Normalized transmission spectra (by channel width, in Å) along each direction for (b) metallic and (c) the multiradical electronic solution of BPN. We utilized 2 × 2 supercells for these calculations, as schematically shown in (a). Note that spin-up/spin-down transmission curves are perfectly superimposed for the multiradical case. Bond current maps of injected currents (*E* – *E*_F_ = 0.5 eV) in large-scale devices made of BPN in (d) the metallic state and (e) the multiradical state. Areas with high current density are shown in bright yellow, and those with low or no current density are depicted in dark purple (see color bars). (f) Net spin polarization map of bond currents (spin-up: blue and spin-down: red) for the BPN device in the multiradical state and zoomed view around the injection site (in yellow). Small red dots at the bottom of each large-scale device indicates the point of injection (d,e,f).

To gain insights into this, we have carried out transport calculations based on the Green’s function method (see the Experimental Section for details) for each of the two possible electronic solutions of BPN; namely, the non-spin-polarized state and the multiradical one. We note that an initial AFM spin guess had to be used in order to obtain the multiradical state, as explained above. As schematically represented in Figure 3a, we first construct small devices that allow us to compute the electronic transmission along each in-plane direction. We normalize the transmission by the width of each channel, to ensure that the comparison is independent of our unit cell choice. As can be seen in Figure 3b,c, BPN displays a preference for electron transport through the CBD-like unit (*y*-direction) rather than through the single C–C bonds connecting the hexagonal rings (*x*-direction), for both electronic solutions. This goes in line with prior theoretical results showing the metallic character of hexagonal rings when connected via CBD-like square units^37^ and suggests that BPN may behave as an array of weakly coupled 1D BP^38^ channels.

To assess such a hypothesis, we simulate the spatial distribution of injected currents in large-scale devices made of BPN, modeled with DFT-parametrized tight binding (TB) Hamiltonians, which capture the low-energy spectra of the material (see the Experimental Section for details). Figure S6 shows the benchmark of such DFT-parametrized TB models separately for each electronic solution. As seen in Figure 3d,e, injected currents in BPN mainly propagate along the *y*-direction (i.e., through the BP 1D wires) for both the metallic and multiradical electronic configurations. However, to a minor degree, they also spread along the *x*-direction, which arises from the electronic coupling between the 1D channels. The resulting conic-shaped bond-current distribution is known as the Talbot interference pattern; a key characteristic of optical waveguides,^53,54^ which has also been recently reported for electronically anisotropic carbon 2D materials that behave as 2D arrays of nanoelectronic 1D channels.^50^ Thus, the results, as shown in Figure 3, confirm that BPN belongs to this novel class of nanostructured carbon 2D materials. Finally, if we look at the normalized difference of bond currents between the two spin channels for the multiradical solution (Figure 3f), we see that injected currents are strongly spin-polarized in this state. This stems from the local spin polarization at the particular injection site (see the atomic position in yellow in the zoomed view of Figure 3f), and so injecting at a different position changes the sign and degree of spin polarization (see Figure S7). Experimentally, such a spintronic effect could be realized by injecting currents with a magnetically polarized Fe-functionalized scanning tunneling microscopy tip.^55^

## Conclusions

To conclude, our theoretical studies combining first principles DFT calculations with quantum transport simulations have revealed key electronic properties of the BPN. By establishing a connection with molecular BP, and in particular with CBD, we have demonstrated the emergence of the radical character in the square rings embedded in BPN. Our findings thus demonstrate that BPN displays a spin-polarized multiradical ground state, which was missed in prior studies on such 2D organic material.^21−25^ A similar situation was previously reported for post-graphene organic Dirac materials (e.g., graphynes^8^) where low-lying multiradical states were also missed,^13^ which highlights the need to consider such type of multiradical spin-polarized configurations when modeling carbon-based metallic (or semimetallic) 2D materials. As we mentioned above, a proper spin-polarized initial guess is necessary to capture such multiradical electronic solutions in DFT. By running AIMDS, we have found that the multiradical character emerges spontaneously under thermal fluctuations (i.e., it is thermodynamically stable), in agreement with other studied multiradical 1D^56^ and 2D organic polymers.^41,57^ Despite the fact that our tested DFT functionals are able to capture this multiradical state, they disagree about the corresponding band gap, so that no conclusive semiconductor/metallic behavior can be associated to it. This may be indicative of an electronic structure (band gap) that is sensitive to and tunable by external means such as mechanical strain, doping, nonequilibrium currents, or chemical functionalization. Finally, quantum transport simulations demonstrate the anisotropic character of electronic transport in BPN. The spatial spreading of injected currents follows a Talbot interference pattern, which is a key feature of carbon 2D materials behaving as arrays of weakly coupled 1D nanoelectronic channels.^11,50^ Additionally, this feature appears for both the non-spin-polarized metallic and multiradical states of BPN, suggesting that transport anisotropy is intrinsically linked to the structural in-plane anisotropy of the 2D material. Due to the spin-polarized character of the multiradical configuration, we find a strong spin-filtering effect of injected currents, which could potentially serve for spintronic applications.^58^

Overall, our study provides new insights into fundamental characteristics of BPN which are key for its applicability in future technologies. The herein revealed multiradical nature of BPN has important consequences regarding the chemical stability of this novel 2D organic material. This highlights the need to seek for strategies to stabilize it upon integration in solid-state devices via, for instance, encapsulation in van der Waals heterostructures (e.g., between inert hBN layers^46^). On the other hand, knowledge about the transport properties of BPN is also essential for its applicability in carbon electronics, which is one of the main technological areas where this material bears particular potential.^20^

## Experimental Section

Periodic DFT calculations have been used to optimize both the atomic and electronic structure of BPN. Different hybrid exchange correlation functionals within the GGA^59^ that were tested include HSE06^40^ and PBE0.^42^ We note that hybrid functionals have been shown to properly reproduce experimentally measured magnetic coupling coefficients for carbon-based multiradical oligomers,^41,60^ as well as to capture low-lying multiradical states for 1D^38^ and 2D^13^ carbon nanomaterials (later on experimentally reported^16^), which are missed when using pure GGA^61^ or LDA^12^ approaches. The non-spin-polarized metallic solution was obtained via spin-restricted calculations, whereas the multiradical solution is generated via spin-unrestricted calculations and setting an antiparallel spin initial guess. Both the atomic coordinates and in-plane cell vectors are optimized separately for each electronic solution and DFT functional. We use a tier-1 light numerical atom-centered orbital (NAO) basis set,^62^ as implemented in the Fritz Haber Institute ab initio molecular simulations package (FHI-AIMS).^63,64^ Optimizations for the primary unit cell (2 × 2 supercell) were carried out employing a 30-30-1 (15-15-1) Γ-centered Monkhorst Pack (MP) k-grid, using convergence criteria set to 10^–5^ eV for the total energy and 10^–2^ eV/Å for the maximum force component per atom. Optimized cell vectors and atomic coordinates for each electronic solution, using HSE06, are provided in the Supporting Information. Band structures and atomically partitioned spin populations (using the Hirschfeld method^65^) are generated in a following single-point calculation over the fully optimized structures, using a 100-100-1 (50-50-1) Γ-centered MP k-grid for the primary (2 × 2) unit cell. Other quantities such as total energies and electronic band gaps are extracted from these last single-point calculations. AIMDS are run in FHI-AIMS for each tested DFT functional at 300 K for 4 ps, using the Bussi–Donadio–Parrinello thermostat,^66^ a 6-6-1 Γ-centered MP k-grid, and a Tier-1 light NAO basis set. These AIMDS are carried out in a spin-unrestricted configuration, setting an initial spin guess of 0.

In order to run the transport simulations, we obtained the multiradical and metallic electronic solutions of BPN within the Siesta DFT package. Here, we utilized the PBE functional, a single-ζ basis set with 0.02 Ry energy shift, norm-conserving Troullier–Martins pseudopotentials, and a real-space mesh cutoff of 400 Ry. The same convergence criteria and MP k-grids used in FHI-AIMS calculations were utilized to optimize both the atomic and electronic structure of BPN within Siesta. Transport simulations were modeled by extracting the onsite and coupling elements of p_*z*_ orbitals from the converged DFT Hamiltonians and overlap matrices, to parametrize efficient TB models (see DFT benchmark in Figure S6), which was carried out using the Python-based open-source SISL code.^67^ Transmission along *x* (*y*) was obtained by repeating the BPN 2 × 2 supercell three times along each direction (see Figure 3a) using 350 (200) k-points along the transverse periodic direction. Large-scale BPN devices were built by tiling the unit cell along both in-plane directions until obtaining a sample length of approximately 50 × 45 nm^2^, leading to devices composed of more than 79,000 atoms. The TBtrans code,^68^ based on the Green’s function formalism,^69,70^ was used to simulate quantum transport on these large devices. 2D bond-current maps are used to represent the spatial distribution of injected currents, utilizing a color map proportional to the current magnitude flowing from each atom (i.e., high current: bright yellow and low to zero current: dark purple). More details on this type of device calculations can be found elsewhere.^50,51,71^

The application of electrostatic gates was simulated via a fixed plane of charge parallel to the BPN layer and placed 3.5 Å below it, as previously carried out in other studies.^52,72^ This was carried out with the PBE functional, as implemented in the Siesta code. These calculations were carried out using a 2 × 2 unit cell and applying gates, ranging from +1 electron per cell (n-doping) to −1 electron per cell (p-doping), as shown in Figure S5 in Supporting Information. For each tested gate, the atomic structure and cell parameters of the periodic network were fully optimized, thus also capturing the relaxation of the material due to the addition/subtraction of charge.

## Supporting Information Available

Unit cell choice for AFM alignment and 1 × 2 supercell results; band structure for the non-spin-polarized solution using HSE06; band structure for the spin-polarized multiradical solution using HSE06 and PBE0, respectively; values of spin population and band gap during AIMDS using HSE06 and PBE0, respectively; effect of electrostatic gates on BPN multiradical character; benchmark of DFT-parametrized TB models; spin polarization bond current maps for large-scale BPN devices in the multiradical solution upon injecting at different atomic positions; and cell vectors and atomic coordinates for the optimized BPN structure in the non-spin-polarized and spin-polarized solutions, respectively, using HSE06 (PDF)
