# Supplementary material for: Unveiling the Multiradical Character of the Biphenylene Network and Its Anisotropic Charge Transport
Source: J Am Chem Soc. 2022 Apr 27;144(18):8278–85. doi: 10.1021/jacs.2c02178 (PMC9100647; doi:10.1021/jacs.2c02178)
Supplement: Supplementary file 1 — ja2c02178_si_001.pdf [file ja2c02178_si_001.pdf]

Supporting Information

Unveiling the multiradical character of the biphenylene network and its anisotropic charge transport

Isaac Alcón[1,2]*, Gaetano Calogero[3], Nick Papior[4], Aleandro Antidormi[1], Kenan Song[5], Aron W. Cummings[1], Mads Brandbyge[6,7] and Stephan Roche[1,8]*

[1]*Catalan Institute of Nanoscience and Nanotechnology (ICN2), CSIC and BIST, Campus UAB, Bellaterra, 08193 Barcelona, Spain*

[2]*Institut für Chemie und Biochemie, Physikalische und Theoretische Chemie, Freie Universität Berlin, Arnimallee 22, 14195 Berlin, Germany*

[3]*CNR Institute for Microelectronics and Microsystems (CNR-IMM), Zona Industriale, Strada VIII, 5, 95121 Catania, Italy*

[4]*Computing Center, Technical University of Denmark, DK-2800 Kongens Lyngby, Denmark*

[5]*Physical Science and Engineering Division, King Abdullah University of Science and Technology (KAUST), 23955 Thuwal, Saudi Arabia*

[6]*Department of Physics, Technical University of Denmark, DK-2800 Kongens Lyngby, Denmark*

[7]*Center for Nanostructured Graphene (CNG), DK-2800 Kongens Lyngby, Denmark*

[8]*ICREA, Institució Catalana de Recerca i Estudis Avançats, 08070 Barcelona, Spain*

*Corresponding authors: isaac.alcon@icn2.cat, stephan.roche@icn2.cat

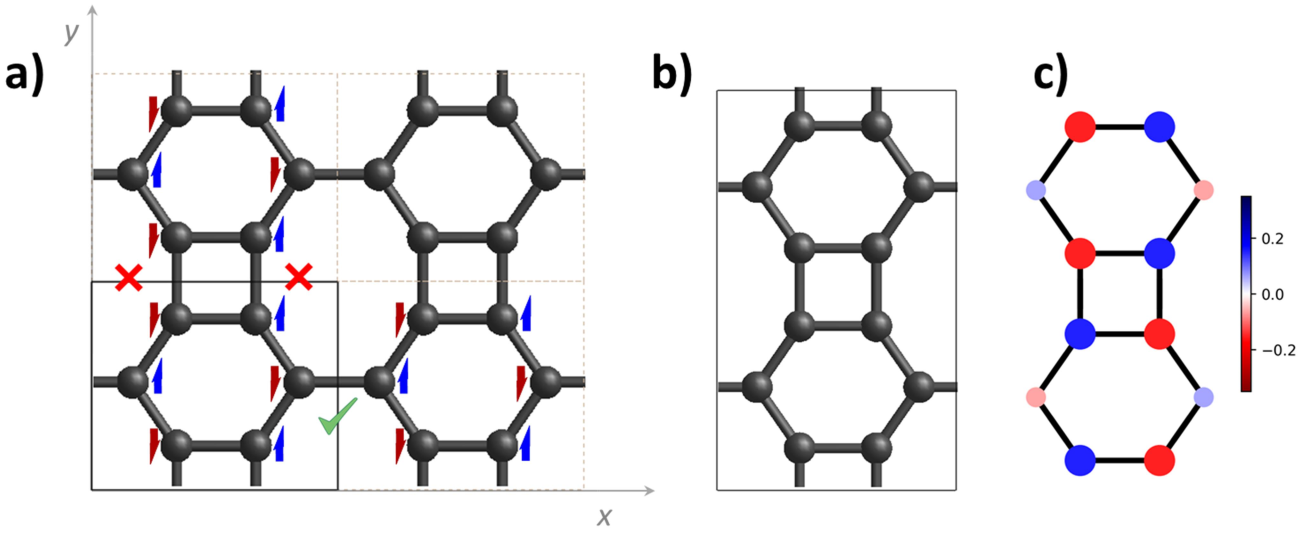

Fig. S1. As sketched in a), the primitive unit cell allows for an antiferromagnetic (AFM) alignment of spins along the *x* direction (even number of carbon atoms), but not along the *y* direction (odd number of carbon atoms). Consequently, the minimal unit cell capable of hosting the AFM ground state of BPN is the 1x2 unit cell, shown in b). c) Atomically resolved spin population map of the multiradical state in such 1x2 BPN cell (spin-up: blue; spin-down: red) obtained from an optimization using the HSE06 DFT functional. The size and colour intensity are proportional to the spin population value.

Table S1. Energy difference between the multiradical (Rad) and the closed-shell metallic (Met) electronic solutions and average of the absolute value of atomic spin populations in the multiradical state ($\langle|\mu_i|\rangle$) as calculated with either HSE06 or PBE0 DFT functionals, considering 1x2 supercells. See the Methods section for details.

	**HSE06**	**PBE0**
**$E_{Rad} - E_{Met}$ (meV)**	-111.9	-199.5
$\langle|\boldsymbol{\mu}_i|\rangle$	0.12	0.14

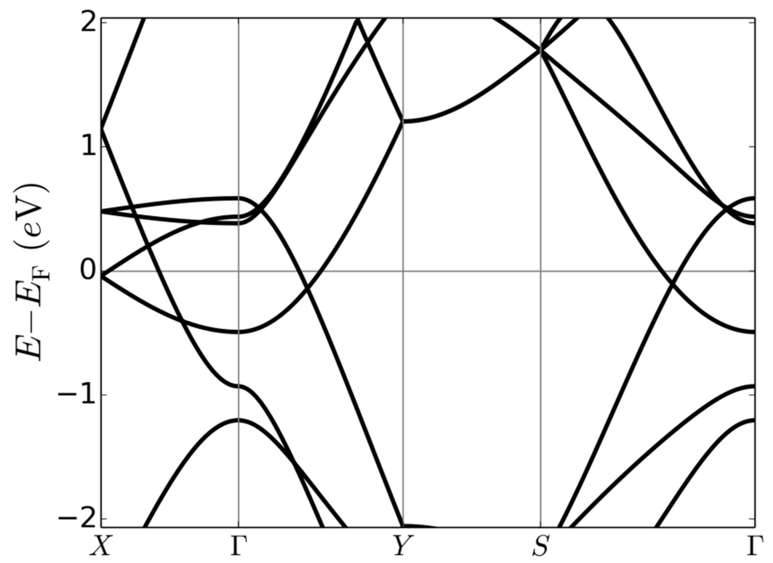

Fig. S2. Band structure in the metallic state of BPN for the 2x2 supercell, obtained using the HSE06 DFT functional.

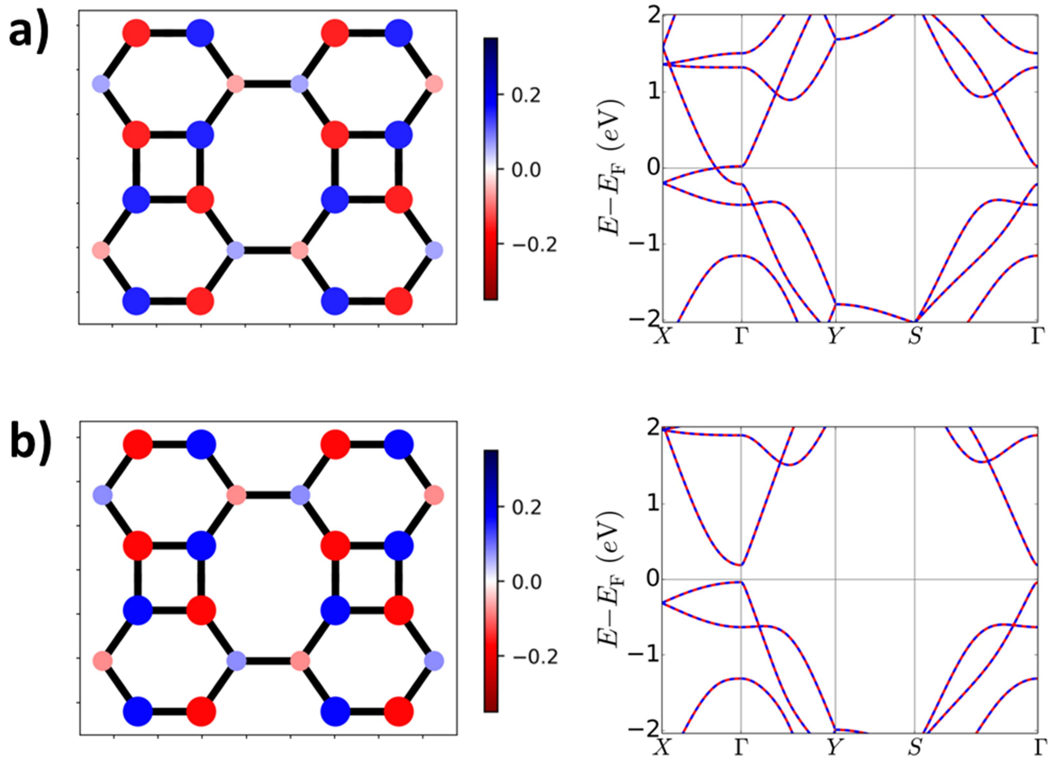

Fig. S3. Atomically resolved spin population maps (left panels) and electronic band structures (right panels) for the multiradical state in BPN as calculated with a) HSE06 and b) PBE0 DFT hybrid functionals. Note that spin-up/spin-down bands are perfectly superimposed.

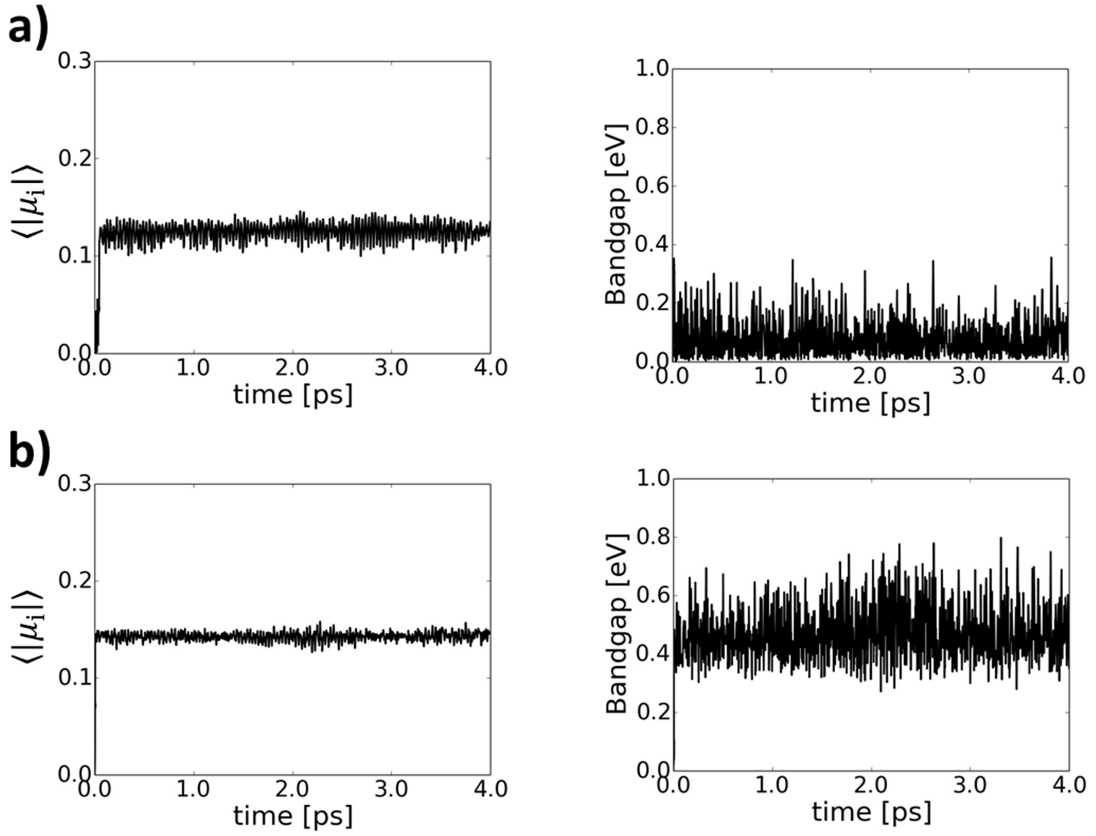

Fig. S4. Average of the absolute atomically-partitioned spin population ($\langle|\mu_{\mathrm{i}}|\rangle$, left panels) and electronic bandgap (right panels) during 4 ps of an ab-initio molecular dynamics simulation of the 2x2 BPN supercell at 300 K using a) the HSE06 and b) the PBE0 functionals.

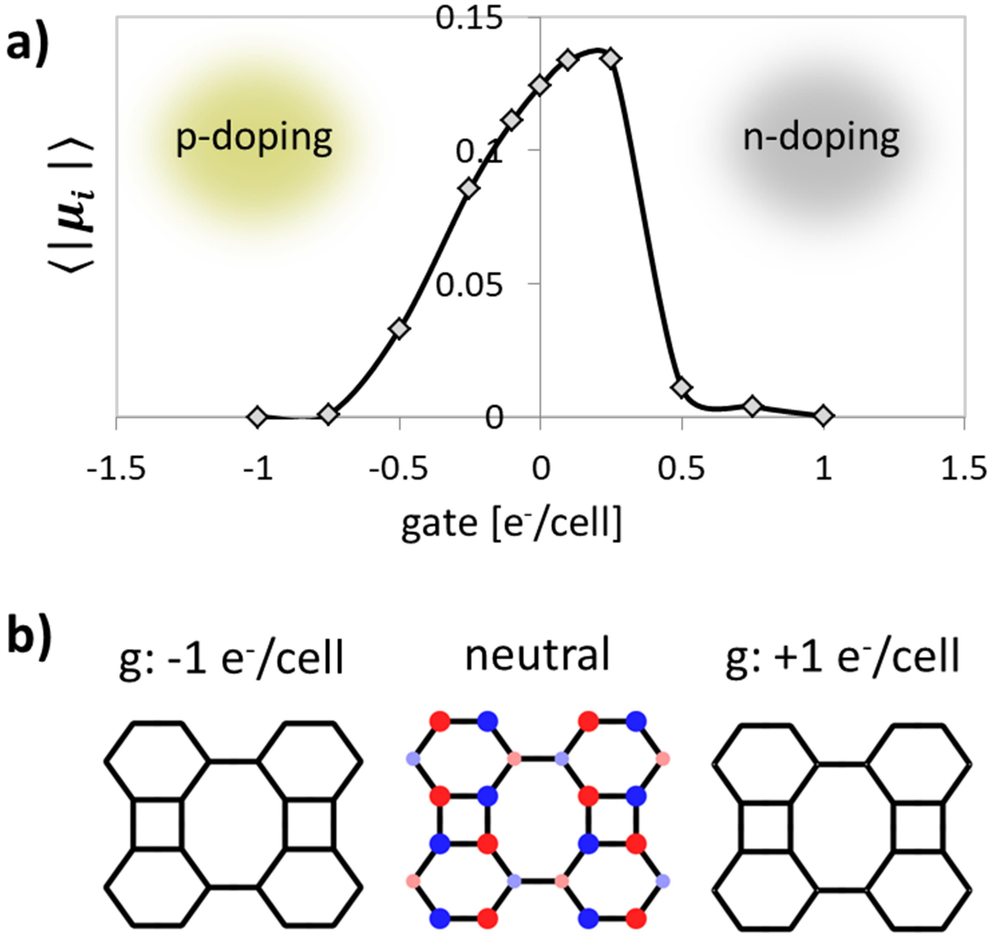

Fig. S5. Effect of electrostatic gates on BPN multiradical character as simulated with the PBE functional (Siesta package) and a 2x2 BPN cell (see Methods section for details). a) Average of the absolute value of atomic spin populations in the multiradical state (⟨|μ_i|⟩) against applied gate, in electrons per 2x2 cell. b) Atomically resolved spin population map in such 2x2 BPN cell (spin-up: blue; spin-down: red) for the three extreme situations (p-doped, neutral and n-doped). The size and colour intensity are proportional to the spin population value.

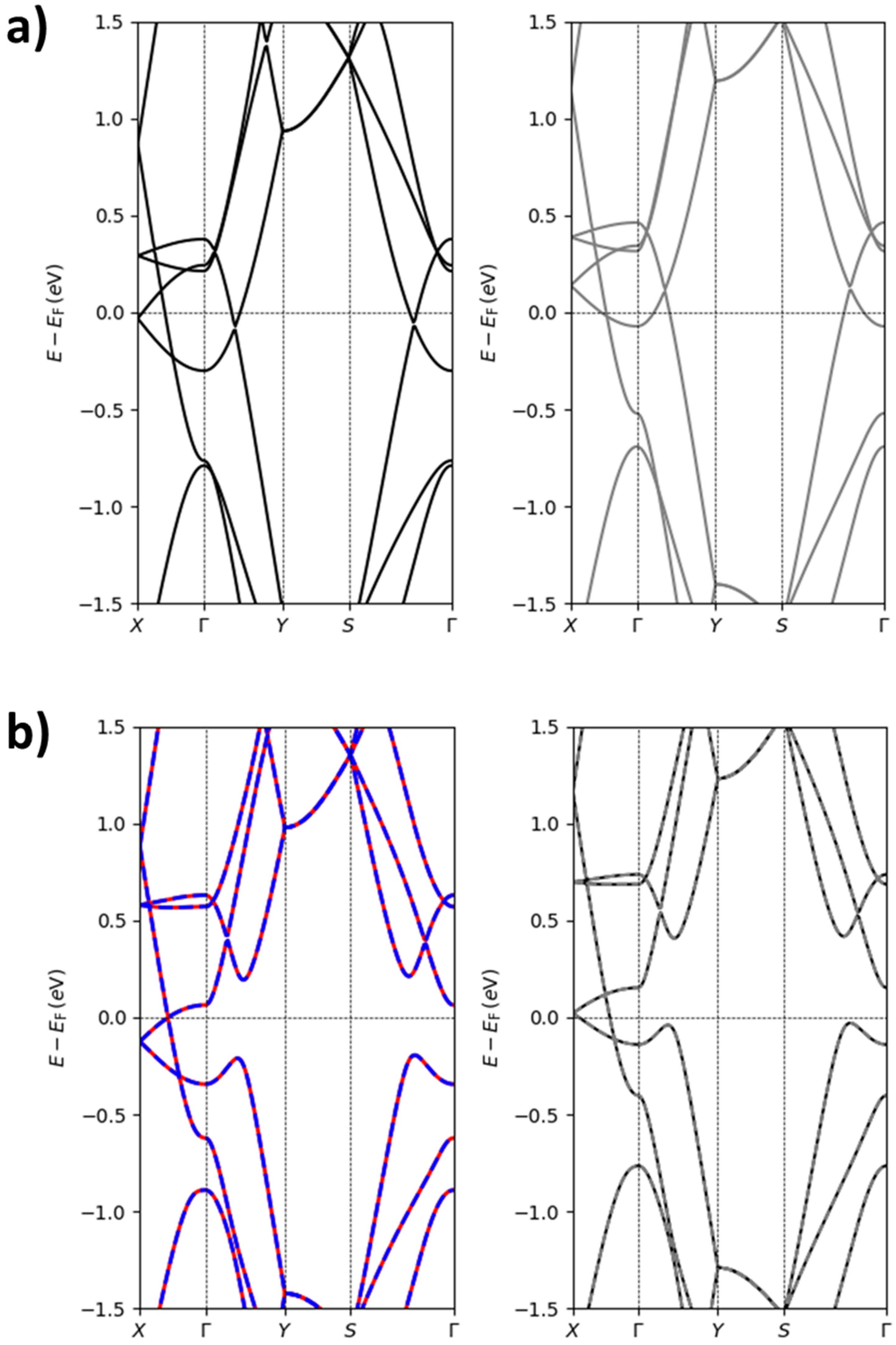

Fig. S6. Comparison between the DFT (left) and DFT-parametrized TB (right) band structures for a) the non-spin polarized metallic and b) the multiradical solutions of BPN. Note that spin-up/spin-down bands are perfectly superimposed in b). Both DFT calculations have been done using a 2x2 supercell and the PBE functional.

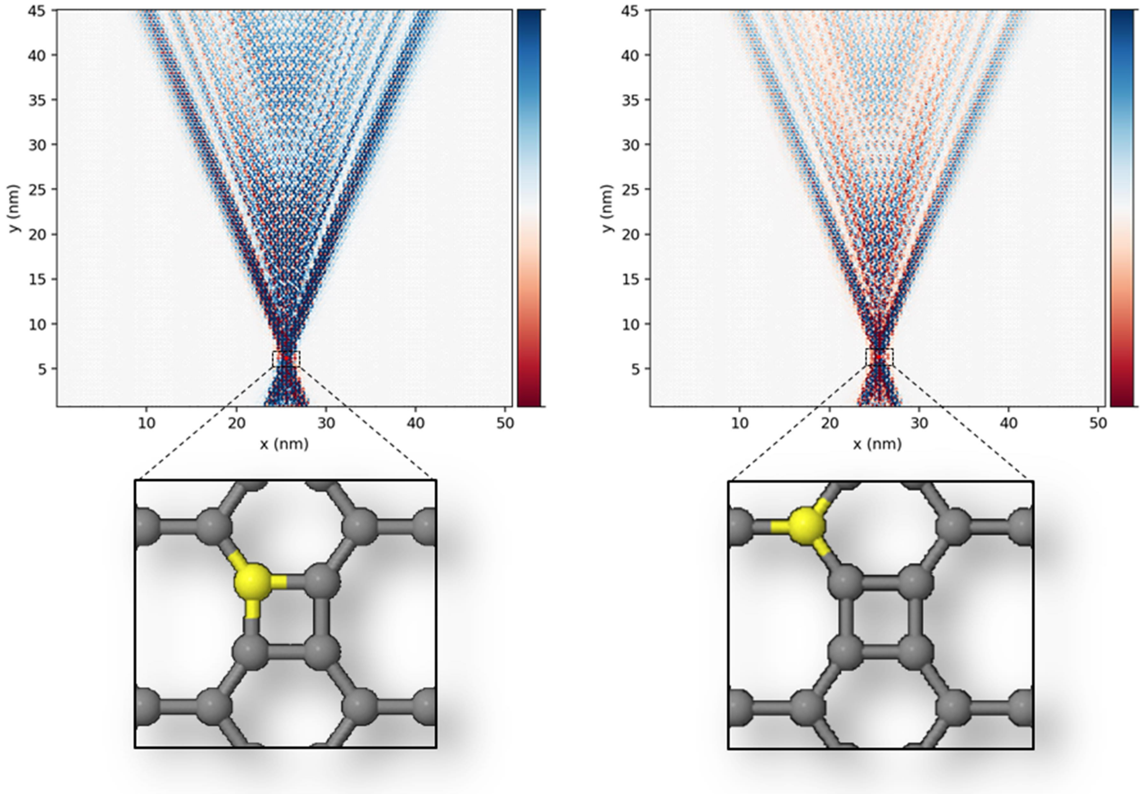

Fig. S7. Net spin polarization map of bond currents (spin-up: blue; spin-down: red) injected in atomic positions within the squared (left) and hexagonal ring (right) of a large-scale BPN device in the multiradical state. Small red dots at the bottom of each device indicates the point of injection, which are also shown (in yellow) in the zoomed atomic views.

Optimized geometries (HSE06 functional) shown in Fig. 2 of the main text (FHI-AIMS format)

Optimized geometry of BPN in the spin-restricted metallic solution (primitive cell)

```
lattice_vector    4.47917648    0.00000000    0.00000000
lattice_vector   -0.00000000    3.73865444    0.00000000
lattice_vector    0.00000000    0.00000000   16.00000000
atom    1.54935725    3.07205284    8.00000000 C
atom    2.98403202    3.07205283    8.00000000 C
atom    0.74839806    1.92979050    8.00000000 C
atom    3.78499125    1.92979049    8.00000000 C
atom    2.98412191    0.78776386    8.00000000 C
atom    1.54926740    0.78776388    8.00000000 C
```

Optimized geometry of BPN in the spin-polarized multiradical solution (super-cell)

```
lattice_vector    8.95649755    0.00000000    0.00000000
lattice_vector    0.00000000    7.48996503    0.00000000
lattice_vector    0.00000000    0.00000000   16.00000000
atom    1.54793490    0.78884582    8.00000000 C
atom    2.98443027    0.78885283    8.00000000 C
atom    3.78401045    1.93300040    8.00000000 C
atom    0.74846349    1.93300035    8.00000000 C
atom    2.98440720    3.07714316    8.00000000 C
atom    1.54795789    3.07715018    8.00000000 C
atom    6.02627635    4.53386557    8.00000000 C
atom    7.46277932    4.53385850    8.00000000 C
atom    8.26224688    5.67797965    8.00000000 C
atom    5.22669991    5.67797963    8.00000000 C
atom    7.46274862    6.82216286    8.00000000 C
atom    6.02630686    6.82215589    8.00000000 C
atom    1.54793103    4.53385848    8.00000000 C
atom    2.98443398    4.53386558    8.00000000 C
atom    3.78401043    5.67797967    8.00000000 C
atom    0.74846351    5.67797961    8.00000000 C
atom    2.98440349    6.82215592    8.00000000 C
atom    1.54796176    6.82216285    8.00000000 C
atom    6.02628006    0.78885281    8.00000000 C
atom    7.46277546    0.78884582    8.00000000 C
atom    8.26224690    1.93300039    8.00000000 C
atom    5.22669991    1.93300035    8.00000000 C
atom    7.46275249    3.07715020    8.00000000 C
atom    6.02630316    3.07714313    8.00000000 C
```
